# Association between heavy metals exposure and persistent infections: the mediating role of immune function

**DOI:** 10.3389/fpubh.2024.1367644

**Published:** 2024-07-22

**Authors:** Huiling Zhang, Juan Wang, Kunlun Zhang, Jianyang Shi, Yameng Gao, Jingying Zheng, Jingtong He, Jing Zhang, Yang Song, Ruifei Zhang, Xuening Shi, Lina Jin, Hui Li

**Affiliations:** School of Public Health, Jilin University, Changchun, China

**Keywords:** heavy metals, heavy metal mixtures, pathogens, infectious disease, immune system

## Abstract

**Introduction:**

Persistent infections caused by certain viruses and parasites have been associated with multiple diseases and substantial mortality. Heavy metals are ubiquitous environmental pollutants with immunosuppressive properties. This study aimed to determine whether heavy metals exposure suppress the immune system, thereby increasing the susceptibility to persistent infections.

**Methods:**

Using data from NHANES 1999–2016, we explored the associations between heavy metals exposure and persistent infections: Cytomegalovirus (CMV), Epstein–Barr Virus (EBV), Hepatitis C Virus (HCV), Herpes Simplex Virus Type–1 (HSV–1), *Toxoplasma gondii* (*T. gondii*), and *Toxocara canis* and *Toxocara cati* (*Toxocara* spp.) by performing logistic regression, weighted quantile sum (WQS) and Bayesian kernel machine regression (BKMR) models. Mediation analysis was used to determine the mediating role of host immune function in these associations.

**Results:**

Logistic regression analysis revealed positive associations between multiple heavy metals and the increased risk of persistent infections. In WQS models, the heavy metals mixture was associated with increased risks of several persistent infections: CMV (OR: 1.58; 95% CI: 1.17, 2.14), HCV (OR: 2.94; 95% CI: 1.68, 5.16), HSV–1 (OR: 1.25; 95% CI: 1.11, 1.42), *T. gondii* (OR: 1.97; 95% CI: 1.41, 2.76), and *Toxocara* spp. (OR: 1.76; 95% CI: 1.16, 2.66). BKMR models further confirmed the combined effects of heavy metals mixture and also identified the individual effect of arsenic, cadmium, and lead. On mediation analysis, the systemic immune inflammation index, which reflects the host’s immune status, mediated 12.14% of the association of mixed heavy metals exposure with HSV–1 infection.

**Discussion:**

The findings of this study revealed that heavy metals exposure may increase susceptibility to persistent infections, with the host’s immune status potentially mediating this relationship. Reducing exposure to heavy metals may have preventive implications for persistent infections, and further prospective studies are needed to confirm these findings.

## Introduction

Heavy metals are a kind of metals and metalloids with density above 4 g/cm^3^, including arsenic (As), cadmium (Cd), cobalt (Co), mercury (Hg), lead (Pb), molybdenum (Mo), antimony (Sb) and tungsten (W) (1). Besides existing widely in the natural environment, heavy metals are widely used in manufacturing, agriculture and homes. Multiple heavy metals are commonly found in daily necessities, such as batteries, paint, cosmetics, and color pigments (2). Humans can be exposed to heavy metals through ingestion, inhalation and dermal absorption (3). In addition, humans can expose to multiple metals simultaneously due to similar exposure routes (2). There may be joint effects between multiple heavy metals on health, such as additive, antagonistic or synergistic effects (4). Due to widespread human exposure and long elimination half–lives, heavy metals have become a concerning class of environmental contaminants.

Existing literature focusing on the impacts of heavy metals on health indicates that exposure to heavy metals could lead to numerous diseases such as cardiovascular disease, nervous system disorders, multiple organ injury and cancers (5, 6). Notably, the potential toxic effect of heavy metals on immune system is receiving increasing attention and being actively researched. Environmental exposure to heavy metals has been noted to disturb immune system and impair its ability against infections (7, 8). Previous epidemiological studies on the impacts of heavy metals exposure on immune system focused on vaccine–induced antibody responses. For instance, prenatal exposure to Cd has been associated with reduced levels of vaccine–induced streptococcal antibodies in the offspring (9, 10). In a cohort study, exposure to Pb was found to be associated with low measles antibody titers in girls, and lower *Haemophilus influenzae* type B titers among children of HIV–positive mothers (11). Exposure to certain heavy metals has also been inversely associated with vaccine–induced diphtheria, pertussis, tetanus, hepatitis B, Japanese encephalitis, polio and measles virus antibodies in children (12). These findings indicate that heavy metals exposure compromises the humoral response, leading to lower vaccine effectiveness.

Currently, numerous pathogens can evade immune clearance to achieve persistent infections, including Cytomegalovirus (CMV), Epstein–Barr Virus (EBV), Hepatitis C Virus (HCV), Herpes Simplex Virus Type–1 (HSV–1), *Toxoplasma gondii* (*T. gondii*), and *Toxocara canis* and *Toxocara cati* (*Toxocara* spp.) (13–16). Recognized as risk factors for all–cause mortality, these infections represent a significant threat to human health, exacerbated by the lack of available vaccines (17). Furthermore, compelling evidence from experimental studies suggests that heavy metals exposure can suppress the innate immunity. Based on these findings, we hypothesize that the immunotoxicity of heavy metals may increase the susceptibility to persistent infections in humans.

In the present study, we included six types of persistent infections: CMV, EBV, HCV, HSV–1, *T. gondii* and *Toxocara* spp. The single and combined effects of multiple heavy metals exposure on these persistent infections were evaluated using logistic regression, weighted quantile regression (WQS), and Bayesian kernel machine regression (BKMR) models. Additionally, this study employed the systemic immune inflammation index (SII) to evaluate the host’s immune status and identified its mediating role in the relationships between heavy metals exposure and the risks of persistent infections. Recognized as a stable and comprehensive biomarker, SII provides an accurate reflection of immune responses and inflammatory conditions (18). Its predictive and prognostic utility has been substantiated in numerous studies (19, 20). Our study might provide a new insight into the impacts of heavy metals exposure on persistent infections.

## Materials and methods

### Study design and population

National Health and Nutrition Examination Survey (NHANES) is a nationally cross–sectional survey conducted by the National Central for Health Statistics. The program aims to estimate the health and nutrition status of residents in the U.S. Participants are interviewed at home and received physical examinations and specimen collection in examination center. National Center for Health Statistics Research Ethics Review Board approved the NHANES survey and all participants provided written informed consents.

In this study, the analyzed data were extracted from public data in the 1999–2016 cycles. We included participants who had measurements of urinary heavy metals and available antibody testing results of CMV, EBV, HCV, HSV–1, *T. gondii* and *Toxocara* spp. Participants with missing data for covariates and mediating variable were excluded. We also excluded pregnant women, as pregnancy could increase susceptibility to infectious diseases (21). Finally, 959, 2,559, 3,725, 4,388, 2,309, and 2,173 participants were left for estimating the association of heavy metals exposure with CMV, EBV, HCV, HSV–1, *T. gondii*, and *Toxocara* spp. infections, respectively. More information about inclusion and exclusion for the study can be found in Supplementary Figure S1.

### Measurement of heavy metals

In 1999–2016 survey years, urinary heavy metals were measured by inductively coupled plasma mass spectrometry technology. Eight elements, including As, Cd, Co, Hg, Pb, Mo, Sb, and W were analyzed in the study. When the concentrations of heavy metals were below the lower limit of detection (LLOD), the values were replaced by LLOD divided by the square root of two. In order to correct urinary dilution on the measurement of urinary heavy metals, urinary creatinine was applied to normalize the concentrations of urinary heavy metals which were expressed as *μ*g/g creatinine.

### Measurement of persistent infections

Available antibody testing results for CMV (1999–2004), EBV (2003–2010), HCV (2007–2012), HSV–1 (2003–2016), *T. gondii* (2009–2012) and *Toxocara* spp. (2011–2014) were examined. With the exception of antibody testing results for EBV, the testing results for other five pathogens were collected from participants aged 18 years or older. For EBV, available serum testing results from subjects aged 6–19 years were determined for EBV VCA IgG antibody using commercial enzyme immunoassay kits. According to the guideline of NHANES, the IgG antibody testing results for these pathogens were defined as positive and negative. The subjects with equivocal testing result were excluded in this study. Detailed laboratory methods can be found on the NHANES website.^1^

### Covariates assessment

All confounders were selected based on prior evidence related to heavy metals exposure and infections. Covariates included gender, age, race/ethnicity, education level, the ratio of family income to poverty (PIR), body mass index (BMI), serum cotinine, smoking status and drinking status. Among them, some covariates were entered into models as continuous variables, including age, PIR, BMI and serum cotinine. The race/ethnicity was divided into non-Hispanic white, non-Hispanic black, Mexican American, other. Education level was categorized into three groups: less than, equal to, and above high school. Smoking status was defined based on whether individuals had smoked at least 100 cigarettes in their lifetime and their current self–reported smoking status. The categories were: never smokers (non-current smoker and smoked less than 100 cigarettes), former smokers (non-current smoker but smoked at least 100 cigarettes) and current smokers (current smoker and smoked at least 100 cigarettes). Based on self–reported daily drinking level, alcohol consumption was defined as never (0 drink for women and men), moderate (≤1 drink for women or ≤2 drinks for men) and heavy (>1 drink for women or >2 drinks for men) (22). Because data on daily alcohol consumption and smoking status are not available for juveniles, the exploration of association between heavy metals exposure and EBV infection was not adjusted for smoking status and alcohol consumption.

### Statistical analysis

The quantitative data was tested for the distribution type using the Kolmogorov–Smirnov test. In this study, the continuous variables were presented with medians and interquartile ranges, as the data were not normally distributed. Categorical variables were presented with *n* (%). The Mann–Whitney *U* test and Chi–square test were used to compare differences of participants characteristics by persistent infections status. The concentrations of heavy metals were ln–transformed due to the seriously skewed distribution. The Spearman rank correlation analysis was applied to calculate the correlation coefficients among ln–transformed concentrations of heavy metals.

We performed multivariable logistic regression models with ln–transformed concentration of each heavy metals as continuous variables to estimate the single effects of heavy metal exposure on the risks of persistent infections.

The WQS regression models were used to evaluate the overall effect of multiple heavy metals exposure on persistent infections. The model estimated an empirically weighted index based on the quantiles of chemicals (23). In the present study, WQS index was created based on quartiles of the heavy metals. The average empirical weights of individual heavy metals were calculated to assess the contributing effects of single heavy metal by using bootstrap sampling. The WQS weight index ranges from 0 to 1, with the sum of all weight index totaling 1. We initially constrained the model in the positive direction based on prior evidence, and subsequently constrained the direction to be negative to determine if there were negative relationships.

We further used the BKMR models to assess the potential associations between heavy metals and persistent infections. Under non-parametric Bayesian variable selection framework, BKMR model can estimate the non-linear and non-additive relationships among mixture exposures (24). Posterior inclusion probability (PIP) was calculated to measure the relative importance of individual exposures and the exposure variable with PIP greater than 0.5 was considered significant (25). Here, we evaluated the effects of heavy metals by using the Markov Chain Monte Carlo method for 10,000 iterations. All covariates were adjusted in BKMR models.

To explore the potential mediating effect of SII, we carried out mediation analyses. SII was calculated using the following formula: platelet count × neutrophil count/lymphocyte count (26). The concentrations of heavy metals mixture were calculated by WQS models and analyzed as continuous variables in mediation analysis. The proportion of mediated effect was calculated as follows: (indirect effect/total effect) × 100%. Bootstrapping was used for significance testing and the models adjusted for all covariates.

Finally, two sensitivity analyses were conducted. First, participants with extreme values (>90%, or <10%) of urinary heavy metals were excluded. Second, considering the limitation of the WQS model in limiting the direction of all exposure factors and outcome, we conducted qgcomp model. This model can calculate both the positive and negative weight coefficients for each component within the mixture.

All statistical analyses were conducted using SPSS statistical software (version 24.0; SPSS Inc., Chicago, IL, United States) and R software (version 4.2.1) with “gWQS” (version 3.0.4), “bkmr” (version 0.2.2) and “mediation” (version 4.5.0) packages. *P*–value <0.05 (two–tailed) was considered significant.

## Results

### Population characteristics

The basic characteristics of the study population were presented in Table 1. The prevalence of CMV, EBV, HCV, HSV–1, *T. gondii*, and *Toxocara* spp. infection was 62.25, 77.49, 2.41, 62.23, 9.59, and 6.26%, respectively. The median ages of the CMV, EBV, HCV, HSV–1, *T. gondii*, and *Toxocara* spp. infection groups were 36, 13, 48.5, 34, 48, and 47 years, respectively. In the EBV infection group, the population had a low level of BMI and serum cotinine. In the other five infection groups, about half of the population was non-Hispanic white, had a high school degree or above and were never smokers. Race, education level and PIR were significantly different between infected and non-infected participants (*p* < 0.05).

**Table 1:** Baseline characteristics of study population.

Characteristics	CMV infection		EBV infection		HCV infection		HSV-1 infection		*T. gondii* infection		*Toxocara* spp. infection	
	Total (*N* = 853)	*P^c^*	Total (*N* = 2,545)	*P^c^*	Total (*N* = 3,696)	*P^c^*	Total (*N* = 4,239)	*P^c^*	Total (*N* = 2,295)	*P^c^*	Total (*N* = 2,155)	*P^c^*
Age^a^, years	36.00(28.00, 4.300)	<0.001	13.00(10.00, 16.00)	<0.001	48.50(34.00, 63.00)	0.210	34.00(27.00, 42.00)	<0.001	48.00(33.00, 62.00)	<0.001	47.00(32.00, 62.00)	0.024
PIR^a^	2.38(1.24, 4.00)	<0.001	1.51(0.79, 2.99)	<0.001	2.20(1.15, 4.20)	<0.001	2.15(1.11, 4.06)	<0.001	2.13(1.10, 4.12)	0.009	2.20(1.10, 4.34)	0.004
BMI^a^, kg/m^2^	27.47(23.61, 32.12)	0.001	21.00(17.85, 24.92)	<0.001	27.84(24.29, 32.27)	0.984	27.40(23.81, 32.28)	<0.001	27.80(24.20, 32.44)	0.026	27.60(24.00, 32.30)	0.111
Serum cotinine^a^, ng/mL	0.24(0.04, 109.00)	0.410	0.07(0.02, 0.56)	<0.001	0.05(0.02, 31.58)	<0.001	0.12(0.02, 87.60)	0.271	0.05(0.01, 23.20)	0.928	0.04(0.01, 30.30)	0.089
Gender^b^		<0.001		0.046		0.027		0.006		0.153		<0.001
Male	263(30.83%)		1,328(52.18%)		2025(54.79%)		2,254(53.17%)		1,262(54.99%)		1,175(54.52%)	
Female	590(69.17%)		1,217 (47.82%)		1,671(45.21%)		1985(46.83%)		1,033(45.01%)		980(45.48%)	
Race/ethnicity^b^		<0.001		<0.001		0.004		<0.001		0.075		<0.001
Non-Hispanic White	434(50.88%)		715(28.09%)		1797(48.62%)		1892(44.63%)		1,106(48.19%)		1,011(46.91%)	
Non-Hispanic Black	182(21.34%)		740(29.08%)		746(20.18%)		831(19.60%)		454(19.78%)		419(19.44%)	
Mexican American	174(20.40%)		790(31.04%)		518(14.02%)		769(18.14%)		304(13.25%)		249(11.55%)	
Other	63(7.39%)		300(11.79%)		635(17.18%)		747(17.62%)		431(18.78%)		476(22.09%)	
Education level^b^		<0.001		0.002		<0.001		<0.001		0.001		<0.001
<High School	173(20.28%)		2,318(91.08%)		912(24.68%)		850(20.05%)		513(22.35%)		413(19.16%)	
High School/GED	234(27.43%)		138(5.42%)		835(22.59%)		988(23.31%)		502(21.87%)		459(21.30%)	
>High school	446(52.29%)		89(3.50%)		1949(52.73%)		2,401(56.64%)		1,280(55.77%)		1,283(59.54%)	
Smoking status^b^		0.543		NA		<0.001		0.148		0.101		0.593
Never	436(51.11%)		NA		1794(48.54%)		2,344(55.30%)		1,138(49.59%)		1,120(51.97%)	
Former	138(16.18%)		NA		1,046(28.30%)		699(16.49%)		641(27.93%)		566(26.26%)	
Current	279 (32.71%)		NA		856(23.16%)		1,196(28.21%)		516(22.48%)		469(21.76%)	
Alcohol consumption^b^		0.011		NA		<0.001		<0.001		0.693		0.009
Never	135(15.83%)		NA		761(20.59%)		530(12.50%)		428(18.65%)		401(18.61%)	
Moderate	282(33.06%)		NA		1,397(37.80%)		1,371(32.34%)		865(37.69%)		841(39.03%)	
Heavy	436(51.11%)		NA		1,538(41.61%)		2,338(55.15%)		1,002(43.66%)		913(42.37%)	

BMI, body mass index; CMV, Cytomegalovirus; EBV, Epstein–Barr Virus; GED, general educational development; HCV, Hepatitis C Virus; HSV-1, Herpes Simplex Virus Type-1; NA, not available; PIR, family income to poverty ratio; *T. gondii, Toxoplasma gondii*; *Toxocara* spp., *Toxocara canis* and *Toxocara cati*. ^a^Continuous variables were presented as weighted median (P_25_, P_75_). ^b^Categorical variables were presented as sample number (percentage). ^c^*p*-value were derived from Mann–Whitney U test for continuous variables and Chi-square test for categorical variables.

### Distribution of urinary metals and correlations

The distribution information about urinary heavy metals is exhibited in Supplementary Table S1. The detection frequency of Mo was nearly 100%, which was the highest detection frequency. Mo had the highest and Sb had the lowest concentrations in urinary heavy metals. And the Spearman’s rank correlation coefficient illustrated the relatively weak correlations between heavy metals (Supplementary Figure S2).

### Associations of heavy metals exposure with CMV infection

The associations between individual heavy metal exposure and persistent infections based on multivariable logistic regression models were presented in Table 2. Each one–fold increase in ln–transformed urinary Cd was associated with 43% (OR: 1.43, 95% CI: 1.11, 1.84) higher odds of CMV infection.

**Table 2:** Odd ratios and 95% confidence intervals for associations between heavy metals and persistent infections using multivariable logistic models^a^.

	CMV infection^b^	EBV infection^c^	HCV infection^b^	HSV-1 infection^b^	*T. gondii* infection^b^	*Toxocara* spp. infection^b^
Arsenic	NA	0.99(0.87, 1.13)	1.04(0.82, 1.30)	**1.10(1.03, 1.18)**	**1.22(1.06, 1.40)**	**1.27(1.06, 1.51)**
Cadmium	**1.43(1.11, 1.84)**	1.00(0.85, 1.17)	**2.41(1.70, 3.41)**	**1.25(1.13, 1.39)**	**1.28(1.03, 1.61)**	1.20(0.93, 1.55)
Cobalt	1.10(0.84, 1.45)	0.92(0.77, 1.11)	**1.38(1.01, 1.89)**	1.01(0.90, 1.14)	1.17(0.95, 1.44)	1.06(0.79, 1.42)
Mercury	0.85(0.72, 1.00)	1.09(0.97, 1.21)	0.95(0.73, 1.23)	**1.10(1.03, 1.18)**	1.17(0.99, 1.37)	0.92(0.76, 1.12)
Molybdenum	0.97(0.76, 1.24)	0.85(0.70, 1.02)	1.07(0.77, 1.50)	0.93(0.84, 1.04)	1.17(0.94, 1.47)	1.17(0.89, 1.55)
Lead	1.10(0.85, 1.43)	**1.45(1.23, 1.71)**	1.36(0.99, 1.87)	**1.25(1.13, 1.39)**	**1.55(1.25, 1.91)**	1.28(0.99, 1.64)
Antimony	0.87(0.69, 1.11)	**1.24(1.04, 1.48)**	1.24(0.91, 1.70)	**1.15(1.04, 1.27)**	1.14(0.92, 1.40)	0.89(0.68, 1.17)
Tungsten	0.87(0.72, 1.06)	1.00(0.89, 1.13)	1.25(0.98, 1.60)	0.96(0.89, 1.03)	0.85(0.71, 1.02)	0.88(0.71, 1.09)

CMV, Cytomegalovirus; EBV, Epstein–Barr Virus; HCV, Hepatitis C Virus; HPV, Human papillomavirus; HSV-1, Herpes Simplex Virus Type-1; NA, not available; *T. gondii*: *Toxoplasma gondii*; *Toxocara* spp., *Toxocara canis* and *Toxocara cati*. ^a^Confidence intervals that do not overlap the null value of odd ratio = 1 are shown in blod. ^b^Covariates adjusted include gender, age, race, education level, family income to poverty ratio, body mass index, serum cotinine, smoking status and alcohol consumption. ^c^Covariates adjusted include gender, age, race, education level, family income to poverty ratio, body mass index and serum cotinine.

**Figure 1:**
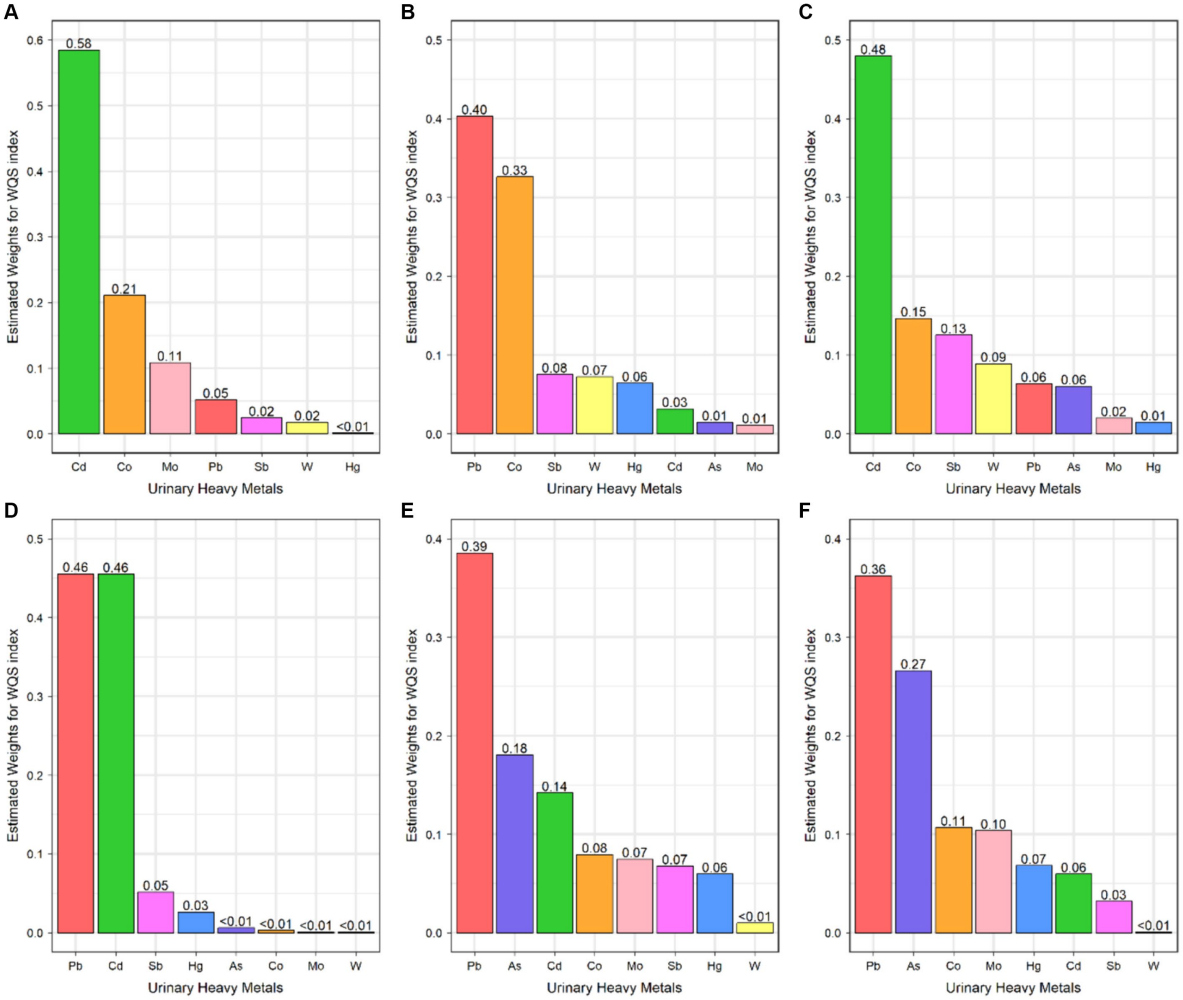
Weighted quantile sum regression index weights for Cytomegalovirus infection **(A)**, Epstein–Barr Virus infection **(B)**, Hepatitis C Virus infection **(C)**, Herpes Simplex Virus Type-1 infection **(D)**, *Toxoplasma gondii* infection **(E)**, and *Toxocara* spp. infection **(F)**.

The WQS analysis was performed to estimate the effects of exposure to heavy metals mixture on the risks of persistent infections. After adjusting all confounders, exposure to heavy metals mixture was significantly positively associated with CMV infection (OR: 1.58, 95% CI: 1.17, 2.14) (Table 3). The estimated weights of heavy metals are displayed in Figure 1A. Cd had the highest weight, with a weight of 0.58. Whereas Hg (weighted <0.01) weighted lightest in CMV infection. In addition, when the direction in WQS models was constrained to be negative, mixed exposure to heavy metals had no significant negative correlations with CMV infection.

**Table 3:** Associations between weighted quantile sum regression index and persistent infections.

Model^a^	Outcomes	OR (95% CI)	*P-*value
Positive			
	Cytomegalovirus infection	1.58(1.17, 2.14)	0.003
	Epstein–Barr Virus infection	1.16(0.94, 1.43)	0.166
	Hepatitis C Virus infection	2.94(1.68, 5.16)	<0.001
	Herpes Simplex Virus Type-1 infection	1.25(1.11, 1.42)	<0.001
	*Toxoplasma gondii* infection	1.97(1.41, 2.76)	<0.001
	*Toxocara* spp. infection	1.76(1.16, 2.66)	0.008
Negative			
	Cytomegalovirus infection	1.15(0.87, 1.52)	0.338
	Epstein–Barr Virus infection	1.06(0.84, 1.33)	0.626
	Hepatitis C Virus infection	0.98(0.63, 1.51)	0.919
	Herpes Simplex Virus Type-1 infection	1.06(0.93, 1.21)	0.373
	*Toxoplasma gondii* infection	0.98(0.78, 1.24)	0.894
	*Toxocara* spp. infection	0.94(0.69, 1.28)	0.682

OR, odds ratio; CI, confidence interval. ^a^ “Negative” and “Positive” indicate the index in weighted quantile sum regression models was restricted in the negative and positive directions.

**Figure 2:**
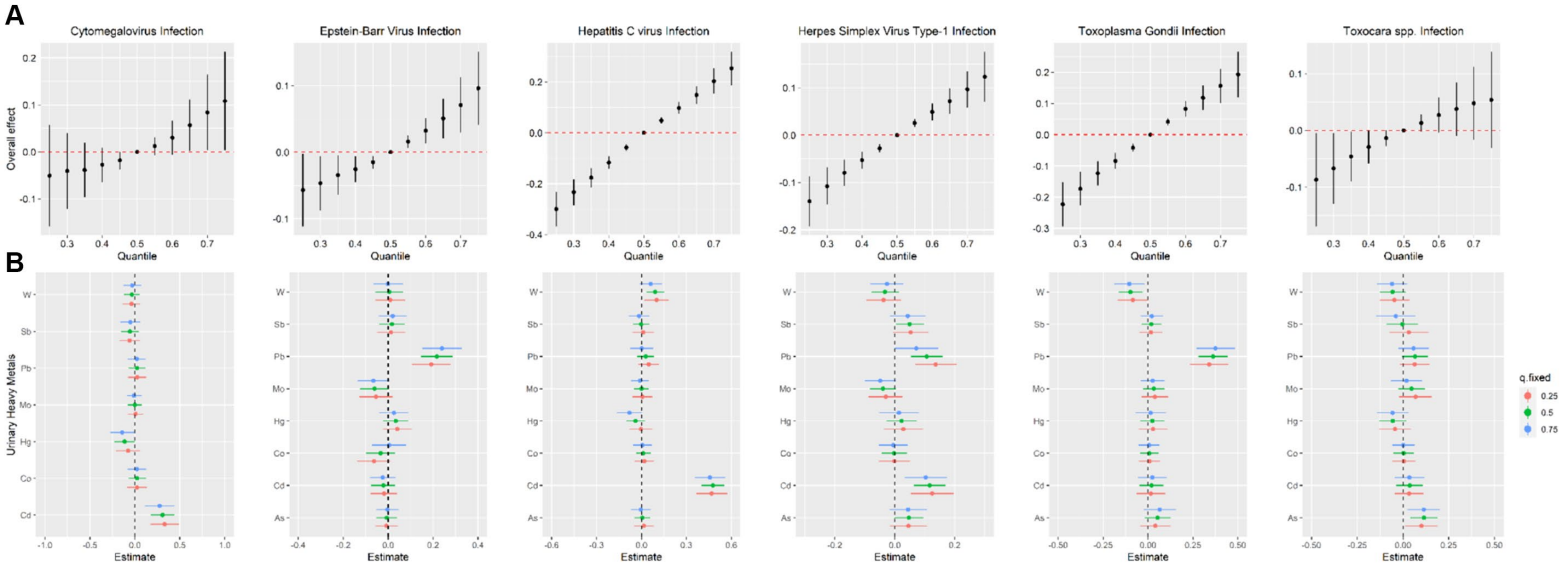
Estimated joint effects **(A)** and independent effects **(B)** of urinary heavy metal mixtures on Cytomegalovirus infection, Epstein–Barr Virus infection, Hepatitis C Virus infection, Herpes Simplex Virus Type-1 infection, *Toxoplasma gondii* infection and *Toxocara* spp. infection by Bayesian kernel machine regression models.

**Figure 3:**
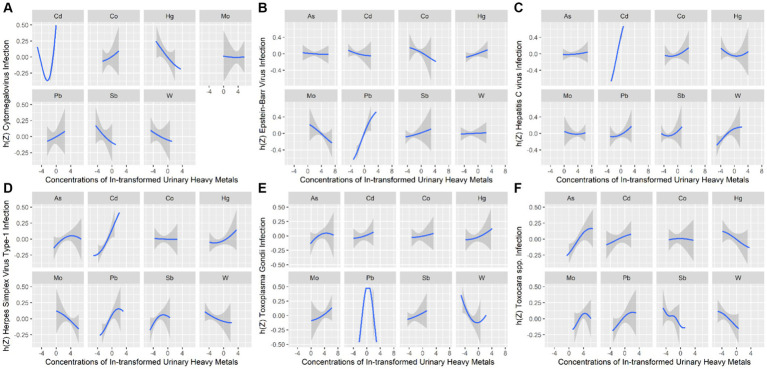
Univariate exposure–response relationships between heavy metals exposure and Cytomegalovirus infection **(A)**, Epstein–Barr Virus infection **(B)**, Hepatitis C Virus infection **(C)**, Herpes Simplex Virus Type-1 infection **(D)**, *Toxoplasma gondii* infection **(E)**, and *Toxocara* spp. infection **(F)**. h(Z) can be interpreted as the relationship between heavy metals and persistent infections.

To further identify the combined effect of heavy metals on persistent infections and potential relationships between heavy metals, we treated the concentrations of heavy metals as continuous variables to fit the BKMR models. The PI*p* values of each heavy metal exposure were summarized in Supplementary Table S2. Results showed that Cd (PIP = 0.996) played a major role in CMV infection. The finding of Cd as the main heavy metal contributing to CMV infection in BKMR models was similar to the result produced by WQS models. Figure 2 represented the joint and independent effects of heavy metals mixture on persistent infections. The latent continuous outcomes of CMV infection showed a significant increase when all heavy metals at 65th percentiles or above in comparison with their median values, indicating significant positive associations between heavy metals mixture and CMV infection (Figure 2A). Cd was significantly related to the increased risk of CMV infection when other heavy metals were fixed at the 25th, 50th, and 75th percentiles (Figure 2B). We also found the non-linear association between Cd and CMV infection when other heavy metals were fixed at their median values (Figure 3A).

### Associations of heavy metals exposure with EBV infection

In multivariable logistic regression model, Pb (OR: 1.45, 95% CI: 1.23, 1.71) and Sb (OR: 1.24, 95% CI: 1.04, 1.48) were positively associated with the risk of EBV infection (Table 2).

As for the combined effect of heavy metals, the significant association between heavy metals mixture and EBV infection in the WQS model was not found (Table 3; Figure 1B).

In BKMR model, Pb appeared to drive the overall effect (PIP = 1.000) (Supplementary Table S2). The joint effect on the risk of EBV infection increased as the cumulative level with heavy metal mixture exposure increased (Figure 2A). Pb displayed a significantly independent effect on the risk of EBV infection, and the association was non-linear (Figures 2B, 3B). Besides, the bivariate exposure–response functions reflecting the interactions between each pair of heavy metals on persistent infections were determined. When Pb was fixed at 10th, 50th, and 90th percentiles, the slope of Co was inconsistent, indicating that there might be interactions between Pb and Co (Supplementary Figure S3B).

### Associations of heavy metals exposure with HCV infection

After adjusting for covariates, the multivariate–adjusted ORs (95% CIs) of HCV risk were 2.41 (1.70, 3.41) for Cd and 1.38 (1.01, 1.89) for Co (Table 2).

In WQS model, a quartile increase in the WQS index of heavy metals mixture was statistically significantly associated with HCV infection (OR: 2.94, 95% CI: 1.68, 5.16) (Table 3). Cd (weighted 0.48) was the most heavily weighted heavy metal responsible for the positive association with HCV infection (Figure 1C).

The PIPs calculated by BKMR model showed that Cd (PIP = 1.000) and W (PIP = 0.555) were found to be relatively important (>0.5) for association between heavy metals mixture and HCV infection (Supplementary Table S2). The overall association between heavy metals mixture and HCV infection is shown in Figure 2A, from which we found a significant and positive association when heavy metals mixture concentration was at 55th percentile and above, compared to their median concentration. When the levels of all heavy metals were below their respective median values, significant negative associations of mixed heavy metals exposure with HCV infection were found, with decreasing negative correlations with increasing exposure levels of heavy metals mixture. Figure 2B displayed the significant and positive effect of Cd on HCV infection, which was consistent with the finding from WQS model. In addition, we found that multiple heavy metals (Co, Hg, Pb, Sb, and W) had non-linear correlations with the risk of HCV infection, with increasing trends in the high concentration (Figure 3C).

### Associations of heavy metals exposure with HSV–1 infection

Multiple heavy metals were associated with the increased odds of HSV–1 infection. The positive associations of As, Cd, Hg, Pb, and Sb with the elevated risks of HSV–1 infection were found and the corresponding ORs and 95% CIs were 1.10 (1.03, 1.18), 1.25 (1.13, 1.39), 1.10 (1.03, 1.18), 1.25 (1.13, 1.39), and 1.15 (1.04, 1.27), respectively (Table 2).

The WQS index of heavy metals mixture was significantly associated with increased odds of HSV–1 infection (OR: 1.25, 95% CI: 1.11, 1.42) (Table 3). Besides, Pb (weighted 0.46) and Cd (weighted 0.46) were the top two weighted heavy metals contributing to the overall effect on HSV–1 infections (Figure 1D).

The BKMR analyses further identified the potential association between heavy metals and HSV–1 infection. In the eight urinary heavy metals, the highest PIP was found for Cd (PIP = 0.990), followed by Pb (PIP = 0.975), Sb (PIP = 0.523), and Mo (PIP = 0.513) (Supplementary Table S2). The concentration of heavy metals mixture at or above the 55th percentile was positively associated with the risk of HSV–1 infection (Figure 2A). Besides, when other heavy metals were fixed at 25th, 50th, and 75th percentile, Cd displayed significant independent effect on the risk of HSV–1 infection (Figure 2B). The trends in exposure–response functions were displayed in Figure 3D. When other heavy metals were fixed at their median values, Cd, Hg, and Pb exhibited non-linear positive relationships with HSV–1 infections. We further investigated the interactions between the heavy metals. The results suggested the existence of potential interactions among As, Cd, Pb, Hg, and Sb (Supplementary Figure S3D).

**Figure 4:**
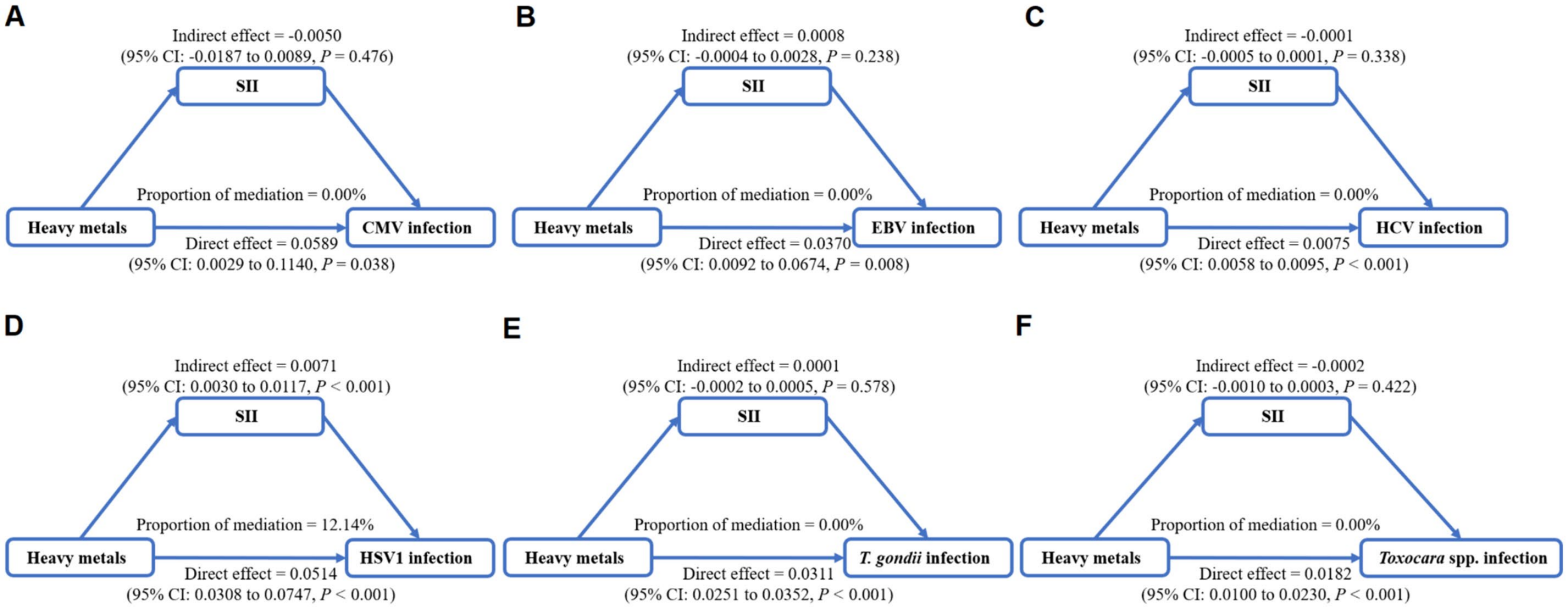
Mediation analysis of systemic immune inflammation index (SII) on the interactions between heavy metals mixture and Cytomegalovirus infection **(A)**, Epstein–Barr Virus infection **(B)**, Hepatitis C Virus infection **(C)**, Herpes Simplex Virus Type-1 infection **(D)**, *Toxoplasma gondii* infection **(E)**, and *Toxocara* spp. infection **(F)**.

To determine if SII, reflecting the host immune status, is a mediator on the associations of heavy metals exposure with persistent infections, we calculated the mixed exposure concentration of eight heavy metals based on WQS models and performed mediation analyses (Figures 4A–F). The significant mediation effect of SII in the association between heavy metals mixture and HSV–1 infections was found. The total effect of heavy metals mixture on HSV–1 infection was 0.0585 and a direct effect of 0.0514. The mediating effect of SII was 0.0071, indicating that SII mediated 12.14% of the association between exposure to heavy metals mixture and HSV–1 infection (Figure 4D).

### Associations of heavy metals exposure with *T. gondii* infection

As for the independent effect of heavy metals, there were significant associations of As, Cd, and Pb with increased odds of *T. gondii* infection, with the adjusted OR (95% CI) of 1.22(1.06, 1.40), 1.28(1.03, 1.61), and 1.55(1.25, 1.91), respectively (Table 2).

Combined exposure to heavy metals was significantly associated with the increased odds of *T. gondii* infection (OR: 1.97; 95% CI: 1.41, 2.76) (Table 3). The highest weighted heavy metal in *T. gondii* infection model was Pb (weighted 0.39). As and Cd were highly weighted in *T. gondii* infection (weighted 0.18 and 0.14, respectively) (Figure 1E).

Similar results were found in BKMR model which identified Pb (PIP = 1.000) as the most important contributor for the positive association with *T. gondii* infection (Supplementary Table S2). Figure 2A showed a significant positive correlation between mixed heavy metals exposure and *T. gondii* infection, suggesting that the risk of *T. gondii* infection will increase significantly when multiple heavy metals are increased. Pb was significantly associated with increased risk of *T. gondii* infection when the concentrations of other heavy metals were fixed at their 25th, 50th, and 75th percentiles (Figure 2B). In univariate exposure–response functions, Co and Sb had approximately linear positive relationships with *T. gondii* infection, and As and Hg exhibited non-linear relationships when each of heavy metals was held at its median value (Figure 3E). Besides, the parallel lines presented in Supplementary Figure S3E indicated no evidence of interaction among these heavy metals.

### Associations of heavy metals exposure with *Toxocara* spp. infection

In logistic regression model, As was found to be significantly associated with *Toxocara* spp. infection (OR: 1.27, 95% CI: 1.06, 1.51) (Table 2).

In the WQS positive model, we found that mixed exposure to eight heavy metals was positively associated with *Toxocara* spp. infection (OR: 1.76, 95% CI: 1.16, 2.66) (Table 3). Pb was the predominant heavy metal responsible for the positive association with *Toxocara* spp. infection (weighted 0.36), followed by As (weighted 0.27) (Figure 1F).

The PIPs derived from the BKMR model showed that all PIP values of eight heavy metals were higher than 0.5, and As (PIP = 0.855), Mo (PIP = 0.692), Pb (PIP = 0.687) had the highest rankings within association between the concentrations of eight heavy metals and *Toxocara* spp. infection (Supplementary Table S2). Although no statistically significant overall associations between heavy metals mixture and *Toxocara* spp. infection were found, there was an increasing trend (Figure 2A). As was significantly related to the risk of *Toxocara* spp. infection when other heavy metals were fixed at 25th, 50th, and 75th percentiles (Figure 2B). When other heavy metals were at the median level, As, Pb, and Cd exhibited positive non-linear association with *Toxocara* spp. infection (Figure 3F).

### Sensitivity analyses

Two sensitivity analyses were conducted to confirm the robustness of our results. The positive associations between heavy metals exposure and persistent infections remained when excluding participants with extreme values of heavy metals (Supplementary Table S3). Besides, qgcomp analysis showed the significant associations of heavy metals mixture exposure with HCV (OR: 2.21; 95% CI: 1.38, 3.56), HSV–1 (OR: 1.29; 95% CI: 1.12, 1.47) and *T. gondii* (OR: 2.03; 95% CI: 1.49, 2.76) infections (Supplementary Table S4 and Supplementary Figure S4).

## Discussion

In this cross–sectional study, we investigated the associations of heavy metals exposure with several persistent infections and the mediating effect of SII which reflected the host immune function in these associations. Using logistic regression, multiple heavy metals (As, Cd, Co, Hg, Pb, and Sb) were identified to be positively associated with the risk of persistent infections. The results of WQS and BKMR analyses consistently showed the association between heavy metals mixture exposure and the increased risk of persistent infections, and emphasized As, Pb, and Cd as the heaviest contributors for persistent infections. In mediation analyses, SII was found to contribute 12.14% in the relationship of heavy metals mixture exposure with HSV–1 infection.

Our findings were supported by previous studies, which suggested associations between higher exposure levels of Pb and Cd and increased risks of *Helicobacter pylori*, herpes simplex virus type 2, hepatitis B virus, *T. gondii* and human immunodeficiency virus infections (27–30). In a prospective study of 214 mother–infant pairs, the higher maternal As exposure was found to relate to infant infections and there was an increasing trend in the number of infant infections with higher maternal As concentrations (31).

The biological mechanisms underlying the association between heavy metals exposure and persistent infections remain inconclusive. A possible situation is that heavy metals exposure affects the risk of infections by altering the immune function. The outcome and severity of infections are highly dependent on innate and adaptive immunity (32). The weakened immune function has been confirmed to be a risk factor for infections. Immunocompromised individuals have been shown to have a higher risk for persistent infections and developing multiple diseases (33, 34). Heavy metals are ubiquitous environmental pollutants with immunotoxicity. Epidemiological investigations found that exposure to heavy metals can suppress the antibody–mediated immunity (35). Data from animal studies suggested that heavy metals exposure also suppresses non-specific immune responses. Exposure to heavy metals can disturb immune function by affecting the production of immune cells, inducing alterations of inflammatory markers, and altering the levels of cytokines (36, 37). These immune substances play an important role in killing virus–infected cells and engulfing parasites (38, 39), and as such, impaired immune function can increase susceptibility to infections. Prior studies have demonstrated that the immunotoxicity of heavy metals could impair the ability to defend infectious agents, subsequently augmenting the host’s susceptibility to infections. Findings by Cox et al. (40) found that exposure to Cd can significantly disrupt the immune function of macrophages, key immune cells that play a pivotal role in the body’s defense against pathogens. This disruption can result in an increased susceptibility to infections, particularly among individuals suffering from chronic obstructive pulmonary disease. Exposure to Pb can exert toxic effects on the immune system, potentially leading to an increased susceptibility to various infections, including Influenza virus and hepatitis B virus (41, 42). A prospective cohort study revealed that prenatal exposure to As in drinking water was associated with an increased risk of developing acute respiratory infection in children (43). Similarly, an experimental study utilizing a mouse model for both prenatal and postnatal periods has discovered that early life exposure to As could aggravate inflammatory responses, thereby elevating the risk of developing respiratory infections (44). Several *in vitro* and experimental studies have also reported similar findings regarding the effects of nickel and Hg (35, 45). Recent studies have revealed a positive correlation between heavy metal exposure and SII, indicating that as the level of heavy metal exposure increases, the body’s immune–inflammatory response intensifies (46–48). Prolonged immune–inflammatory responses can lead to immune system dysregulation and affect the normal functioning of the immune system. Based on a nationally representative sample, we discovered the intermediary role of SII in the interaction of heavy metals exposure with the increased risk of HSV–1 infection, suggesting that the alteration of host immune function might be responsible for the increased risk of persistent infections. The result provided epidemiological evidence for our hypothesis.

In this study, we employed three different statistical methods to explore the association between heavy metals exposure and persistent infections. Logistic regression model is a common analysis approach to assess the effect of single exposure factor on health. Our results of logistic regression analysis showed that typical heavy metals, such as As, Pb, Hg, and Cd, were positively related to several persistent infections. However, relying solely on logistic models might ignore the interactions between heavy metals, contributing to misleading results. WQS and BKMR are two analysis models recently developed to estimate the effects of mixture. By using WQS models, we found that exposure to higher levels of heavy metals mixture could increase the risks of CMV, HCV, HSV–1, *T. gondii* and *Toxocara* spp. infections. Due to the restriction of correlations in one direction, WQS model is unable to evaluate the joint effect of exposure factors in diverse effect directions (49). BKMR is a more comprehensive analysis method for identifying the joint effects of mixture, single exposure effects, non-linear associations, and potential interactions. Besides further confirming the findings in the WQS model, BKMR analysis also found the major contributing effect of As for *Toxocara* spp. infection, Cd for CMV, HCV and HSV–1 infections, and Pb for EBV and *Toxocara* spp. infections. With this approach, we observed the non-linear associations between multiple heavy metals and persistent infections, and potential interactions between heavy metals. These three models estimated the effect of heavy metals on persistent infections from different aspects, verifying the comprehensiveness and reliability of this study.

Several limitations within our study should be mentioned. Firstly, the findings are derived from cross–sectional survey data, which imposes constraints on establishing temporality and causality. Persistent infections are diseases contributed by a variety of factors and developed over a long time. Multiple heavy metals, with bioaccumulation potential, exhibit long half–lives within the human body (50, 51). It remains unclear whether exposure to heavy metals precedes initial infections. Further research employing experimental and prospective design studies are needed to provide more evidence. Secondly, for infections such as CMV (52), EBV (53), HCV (54), HSV–1 (55), *T. gondii* (56), and *Toxocara* spp. (57), IgG antibodies are typically produced within the initial months following infection and generally persist for lifetime of the individual. This timeline may result in the exclusion of very recently acquired infections from our analysis. Thirdly, the accurate assessment of heavy metal exposure is impeded by the body’s metabolism of these substances and the practice of substituting values below LLOD with a fixed value, which may introduce measurement bias. Lastly, our study may not have adequately controlled for all residual confounders that could influence the outcomes, including dietary habits, occupational exposures, and behavioral factors.

## Conclusion

In summary, our results provided evidences for the associations between heavy metals exposure and persistent infections. The typical heavy metals, such as As, Cd, Pb, and Hg, were the main contributors for the increased risk of persistent infections. Furthermore, our study highlighted the mediating role of host immune function in the causal pathway linking mixed heavy metals exposure to an increased risk of HSV–1 infection. Reducing the exposure level of heavy metals could potentially decrease an individual’s susceptibility to infectious diseases and long–term impacts of persistent infections. This study suggested new perspectives for the prevention of persistent infections and provided clues for further studies in this area.

## Data availability statement

Publicly available datasets were analyzed in this study. This data can be found here: https://www.cdc.gov/nchs/nhanes/index.htm.

## Ethics statement

The studies involving humans were approved by National Center for Health Statistics Research Ethics Review Board. The studies were conducted in accordance with the local legislation and institutional requirements. The participants provided their written informed consent to participate in this study.

## Author contributions

HZ: Conceptualization, Data curation, Formal analysis, Methodology, Software, Writing – original draft, Visualization, Writing – review & editing. JW: Conceptualization, Funding acquisition, Methodology, Supervision, Writing – review & editing. KZ: Data curation, Formal analysis, Investigation, Methodology, Software, Validation, Visualization, Writing – original draft. JS: Data curation, Investigation, Methodology, Software, Supervision, Validation, Writing – original draft. YG: Data curation, Methodology, Software, Writing – original draft. JZhe: Investigation, Supervision, Validation, Writing – original draft. JH: Investigation, Supervision, Validation, Writing – original draft. JZha: Investigation, Supervision, Writing – original draft. YS: Investigation, Supervision, Writing – original draft. RZ: Supervision, Writing – original draft. XS: Supervision, Writing – original draft. LJ: Investigation, Methodology, Supervision, Writing – review & editing. HL: Conceptualization, Methodology, Supervision, Writing – review & editing.

## Footnotes

^1^https://www.cdc.gov/nchs/nhanes/index.htm
