# Supplementary material for: Association between heavy metals exposure and persistent infections: the mediating role of immune function
Source: Front Public Health. 2024 Jul 22;12:1367644. doi: 10.3389/fpubh.2024.1367644 (PMC11298456; doi:10.3389/fpubh.2024.1367644)
Supplement: Supplementary file 1 [file Data_Sheet_1.docx]

**Supplemental Material**

**Association between heavy metals exposure and persistent infections: the mediating role of immune function**

**Contents**

**Table S1.** Distribution of urinary heavy metals (*μ*g/L) in study population.

**Table S2.** Posterior inclusion probabilities into models using Bayesian kernel machine regression models.

**Table S3.** Multivariable logistic models to assess the association between heavy metals exposure and persistent infections when further excluding extreme values. ^a^

**Table S4.** Qgcomp regression to assess the association of the mixture of urinary heavy metals with the risks of persistent infections.

**Figure S1.** Flow chart of study population for Cytomegalovirus infection (A), Epstein-Barr Virus infection (B), Hepatitis C Virus infection (C), Herpes Simplex Virus Type-1 infection (D), *Toxoplasma gondii* infection (E) and *Toxocara spp.* infection (F).

**Figure S2.** Spearman correlations matrix among ln-transformed urinary heavy metals in the study populations for Cytomegalovirus infection (A), Epstein-Barr Virus infection (B), Hepatitis C Virus infection (C), Herpes Simplex Virus Type-1 infection (D), *Toxoplasma gondii* infection (E) and *Toxocara spp.* infection (F).

**Figure S3.** Bivariate exposure-response functions for every two metals on Cytomegalovirus infection (A), Epstein-Barr Virus infection (B), Hepatitis C Virus infection (C), Herpes Simplex Virus Type-1 infection (D), *Toxoplasma gondii* infection (E) and *Toxocara spp.* infection (F). h(Z) can be interpreted as the relationship between heavy metals and persistent infections.

**Figure S4.** Qgcomp index weights of each heavy metals for the risks of persistent infections. The model was adjusted for gender, age, race, education level, poverty, BMI, serum cotinine, smoking status and alcohol consumption. Cytomegalovirus infection (A), Epstein-Barr Virus infection (B), Hepatitis C Virus infection (C), Herpes Simplex Virus Type-1 infection (D), *Toxoplasma gondii* infection (E) and *Toxocara spp.* infection (F).

**Table S1.** Distribution of urinary heavy metals (*μ*g/L) in study population.

| **Infection group** |  | As | Cd | Co | Hg | Mo | Pb | Sb | W |
| --- | --- | --- | --- | --- | --- | --- | --- | --- | --- |
| **CMV infection** |  |  |  |  |  |  |  |  |  |
|  | Detection rate | NA | 97.54% | 95.31% | 87.22% | 99.77% | 97.19% | 98.01% | 76.44% |
|  | Median | NA | 0.260 | 0.360 | 0.590 | 45.000 | 0.680 | 0.110 | 0.070 |
|  | Interquartile range | NA | 0.130-0.504 | 0.220-0.590 | 0.260-1.430 | 23.650-72.400 | 0.385-1.105 | 0.060-0.170 | 0.030-0.130 |
| **EBV infection** |  |  |  |  |  |  |  |  |  |
|  | Detection rate | 99.37% | 80.47% | 99.06% | 88.76% | 100.00% | 97.88% | 91.36% | 97.84% |
|  | Median | 7.140 | 0.095 | 0.467 | 0.320 | 65.100 | 0.560 | 0.090 | 0.132 |
|  | Interquartile range | 4.245-12.855 | 0.049-0.170 | 0.305-0.733 | 0.160-0.660 | 37.800-100.000 | 0.320-0.930 | 0.050-0.147 | 0.070-0.242 |
| **HCV infection** |  |  |  |  |  |  |  |  |  |
|  | Detection rate | 98.59% | 93.83% | 99.51% | 94.67% | 99.95% | 97.08% | 69.53% | 86.88% |
|  | Median | 8.340 | 0.252 | 0.333 | 0.400 | 41.800 | 0.520 | 0.053 | 0.074 |
|  | Interquartile range | 4.130-17.518 | 0.128-0.482 | 0.204-0.511 | 0.190-0.84 | 22.325-72.175 | 0.290-0.89 | 0.029-0.090 | 0.036-0.144 |
| **HSV1 infection** |  |  |  |  |  |  |  |  |  |
|  | Detection rate | 98.82% | 90.59% | 98.80% | 83.30% | 99.95% | 97.29% | 78.32% | 88.06% |
|  | Median | 7.820 | 0.176 | 0.374 | 0.340 | 43.540 | 0.410 | 0.059 | 0.077 |
|  | Interquartile range | 3.990-16.190 | 0.085-0.345 | 0.223-0.584 | 0.150-0.750 | 22.000-74.400 | 0.230-0.750 | 0.031-0.105 | 0.035-0.150 |
| ***T. gondii* infection** |  |  |  |  |  |  |  |  |  |
|  | Detection rate | 98.08% | 92.59% | 99.43% | 94.90% | 100.00% | 96.64% | 64.66% | 84.71% |
|  | Median | 8.280 | 0.235 | 0.320 | 0.370 | 40.100 | 0.470 | 0.048 | 0.067 |
|  | Interquartile range | 4.060-18.040 | 0.120-0.453 | 0.196-0.506 | 0.180-0.790 | 21.700-68.800 | 0.270-0.820 | 0.029-0.084 | 0.033-0.131 |
| ***Toxocara spp.* infection** |  |  |  |  |  |  |  |  |  |
|  | Detection rate | 98.52% | 89.93% | 99.63% | 83.29% | 100.00% | 97.35% | 67.10% | 80.56% |
|  | Median | 6.980 | 0.194 | 0.341 | 0.300 | 35.700 | 0.370 | 0.045 | 0.059 |
|  | Interquartile range | 3.470-14.49 | 0.089-0.399 | 0.201-0.569 | 0.140-0.630 | 18.540-63.300 | 0.200-0.640 | 0.029-0.078 | 0.026-0.121 |

Abbreviations: As: arsenic; Cd: cadmium; Co: cobalt; Hg: mercury; Mo: molybdenum; Pb: lead; Sb: antimony; W: tungsten. CMV: Cytomegalovirus; EBV: Epstein-Barr Virus; HCV: Hepatitis C Virus; HSV1: Herpes Simplex Virus Type-1; *T. gondii*: *Toxoplasma gondii*; *Toxocara spp.*: *Toxocara canis* and *Toxocara cati*.

**Table S2.** Posterior inclusion probabilities into models using Bayesian kernel machine regression models.

| **Heavy metals** | **PIP ^a^** | **PIP ^b^** | **PIP ^c^** | **PIP ^d^** | **PIP ^e^** | **PIP ^f^** |
| --- | --- | --- | --- | --- | --- | --- |
| Arsenic | NA | 0.062 | 0.196 | 0.329 | 0.208 | 0.855* |
| Cadmium | 0.996* | 0.153 | 1.000* | 0.990* | 0.113 | 0.629* |
| Cobalt | 0.190 | 0.464 | 0.381 | 0.222 | 0.053 | 0.553* |
| Mercury | 0.315 | 0.167 | 0.463 | 0.301 | 0.122 | 0.643* |
| Molybdenum | 0.125 | 0.388 | 0.274 | 0.513* | 0.130 | 0.692* |
| Lead | 0.126 | 1.000* | 0.426 | 0.975* | 1.000 * | 0.687* |
| Antimony | 0.217 | 0.163 | 0.378 | 0.523* | 0.064 | 0.637* |
| Tungsten | 0.124 | 0.070 | 0.555* | 0.295 | 0.365 | 0.681* |

^a^ PIPs were obtained in BKMR model for urinary metals and Cytomegalovirus infection.

^b^ PIPs were obtained in BKMR model for urinary metals and Epstein-Barr Virus infection.

^c^ PIPs were obtained in BKMR model for urinary metals and Hepatitis C Virus infection.

^d^ PIPs were obtained in BKMR model for urinary metals and Herpes Simplex Virus Type-1 infection.

^e^ PIPs were obtained in BKMR model for urinary metals and *Toxoplasma gondii* infection.

^f^ PIPs were obtained in BKMR model for urinary metals and *Toxocara* spp. infection.

*PIPs were more than 0.5.

**Table S3.** Multivariable logistic models to assess the association between heavy metals exposure and persistent infections when further excluding extreme values. ^a^

|  | **CMV infection ^b^** | **EBV infection ^c^** | **HCV infection ^b^** | **HSV1 infection ^b^** | ***T. gondii* infection ^b^** | ***Toxocara* spp. infection ^b^** |
| --- | --- | --- | --- | --- | --- | --- |
| Arsenic | NA | 1.02(0.84, 1.25) | 0.93(0.67, 1.30) | **1.20(1.09, 1.34)** | **1.23(1.00, 1.52)** | **1.39(1.08, 1.78)** |
| Cadmium | **1.45(1.03, 2.03)** | 0.99(0.82, 1.20) | **2.07(1.27, 3.37)** | **1.21(1.05, 1.38)** | **1.37(1.02, 1.83)** | 1.19(0.84, 1.67) |
| Cobalt | 0.95(0.67, 1.35) | 0.80(0.64, 1.02) | 1.29(0.81, 2.06) | 1.06(0.92, 1.24) | **1.45(1.05, 2.01)** | 1.22(0.83, 1.80) |
| Mercury | 0.80(0.65, 1.00) | 1.07(0.92, 1.23) | 0.86(0.60, 1.23) | **1.09(1.01, 1.20)** | 1.22(0.97, 1.53) | 0.97(0.76, 1.25) |
| Molybdenum | 0.82(0.60, 1.13) | 0.77(0.60, 1.00) | 0.80(0.51, 1.24) | 1.01(0.87, 1.16) | 1.25(0.93, 1.69) | 1.32(0.90, 1.93) |
| Lead | 1.18(0.83, 1.69) | **1.54(1.23, 1.92)** | 1.21(0.75, 1.96) | **1.32(1.16, 1.51)** | **1.96(1.44, 2.67)** | **1.49(1.03, 2.15)** |
| Antimony | 0.80(0.59, 1.09) | 1.24(0.98, 1.56) | 1.27(0.80, 2.02) | **1.22(1.06, 1.39)** | 1.03(0.76, 1.39) | 1.07(0.75, 1.52) |
| Tungsten | 0.80(0.61, 1.04) | 0.93(0.78, 1.11) | **1.53(1.08, 2.18)** | 0.94(0.84, 1.04) | 1.00(0.78, 1.29) | 0.94(0.71, 1.25) |

Abbreviations: CMV: Cytomegalovirus; EBV: Epstein-Barr Virus; HCV: Hepatitis C Virus; HSV1: Herpes Simplex Virus Type-1; TG: *Toxoplasma gondii*; *Toxocara spp.*: *Toxocara canis* and *Toxocara cati*.

^a^ Confidence intervals that do not overlap the null value of odd ratio =1 are shown in blod.

^b^ Covariates adjusted include gender, age, race, education level, family income to poverty ratio, body mass index, serum cotinine, smoking status and alcohol consumption.

^c^ Covariates adjusted include gender, age, race, education level, family income to poverty ratio, body mass index and serum cotinine.

**Table S4.** Qgcomp regression to assess the association of the mixture of urinary heavy metals with the risks of persistent infections.

| **Outcomes** | **OR (95% CI)** | ***P* value** |
| --- | --- | --- |
| Cytomegalovirus infection | 1.04(0.76, 1.43) | 0.803 |
| Epstein-Barr Virus infection | 1.19(0.97, 1.46) | 0.102 |
| Hepatitis C Virus infection | 2.21(1.38, 3.56) | 0.001 |
| Herpes Simplex Virus Type-1 infection | 1.29(1.12, 1.47) | <0.001 |
| *Toxoplasma gondii* infection | 2.03(1.49, 2.76) | <0.001 |
| *Toxocara* spp. infection | 1.44(1.00, 2.09) | 0.053 |


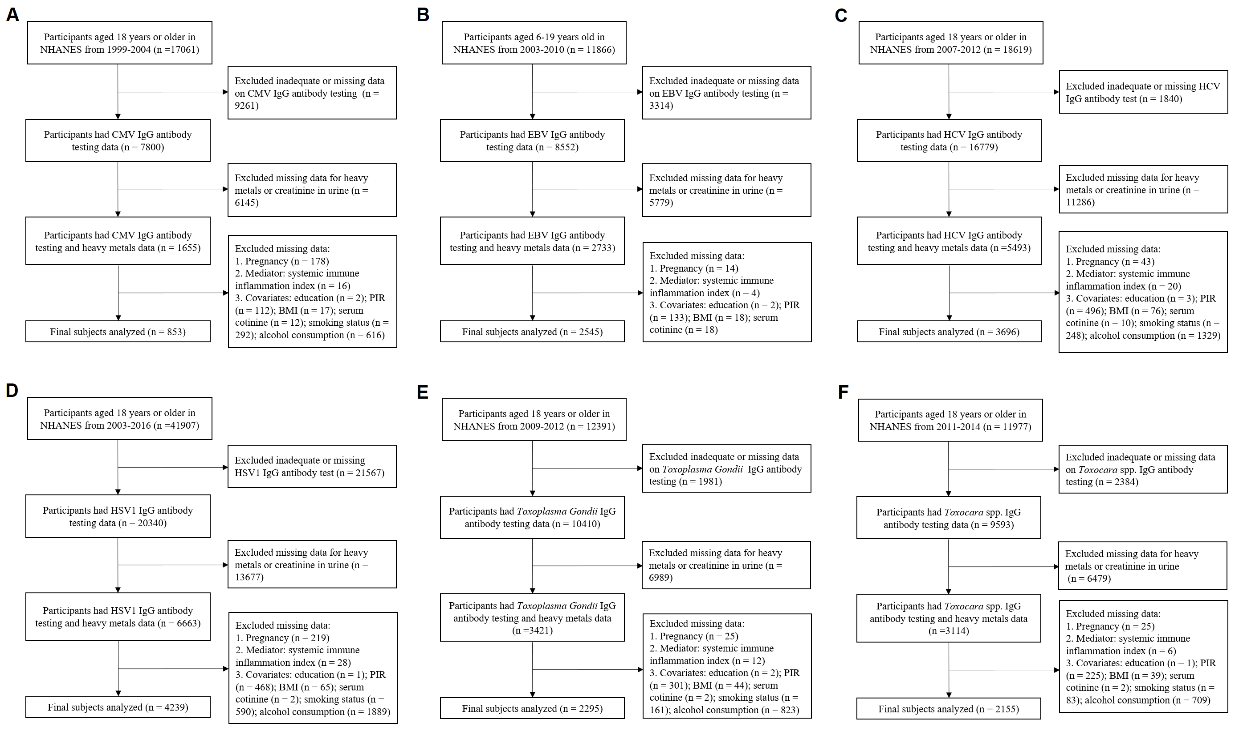


**Figure S1.** Flow chart of study population for Cytomegalovirus infection (A), Epstein-Barr Virus infection (B), Hepatitis C Virus infection (C), Herpes Simplex Virus Type-1 infection (D), *Toxoplasma gondii* infection (E) and *Toxocara spp.* infection (F).


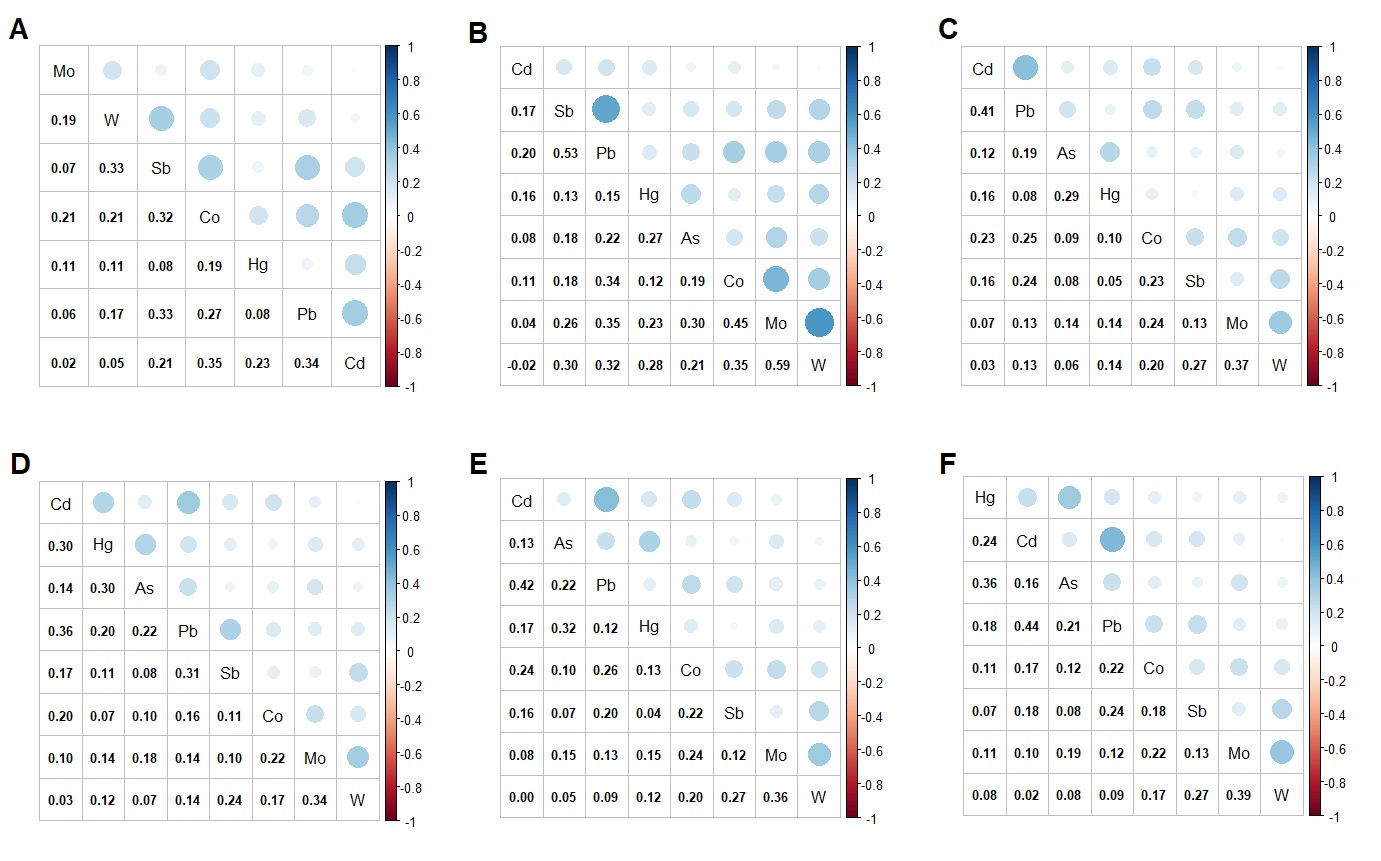


**Figure S2.** Spearman correlations matrix among ln-transformed urinary heavy metals in the study populations for Cytomegalovirus infection (A), Epstein-Barr Virus infection (B), Hepatitis C Virus infection (C), Herpes Simplex Virus Type-1 infection (D), *Toxoplasma gondii* infection (E) and *Toxocara spp.* infection (F).


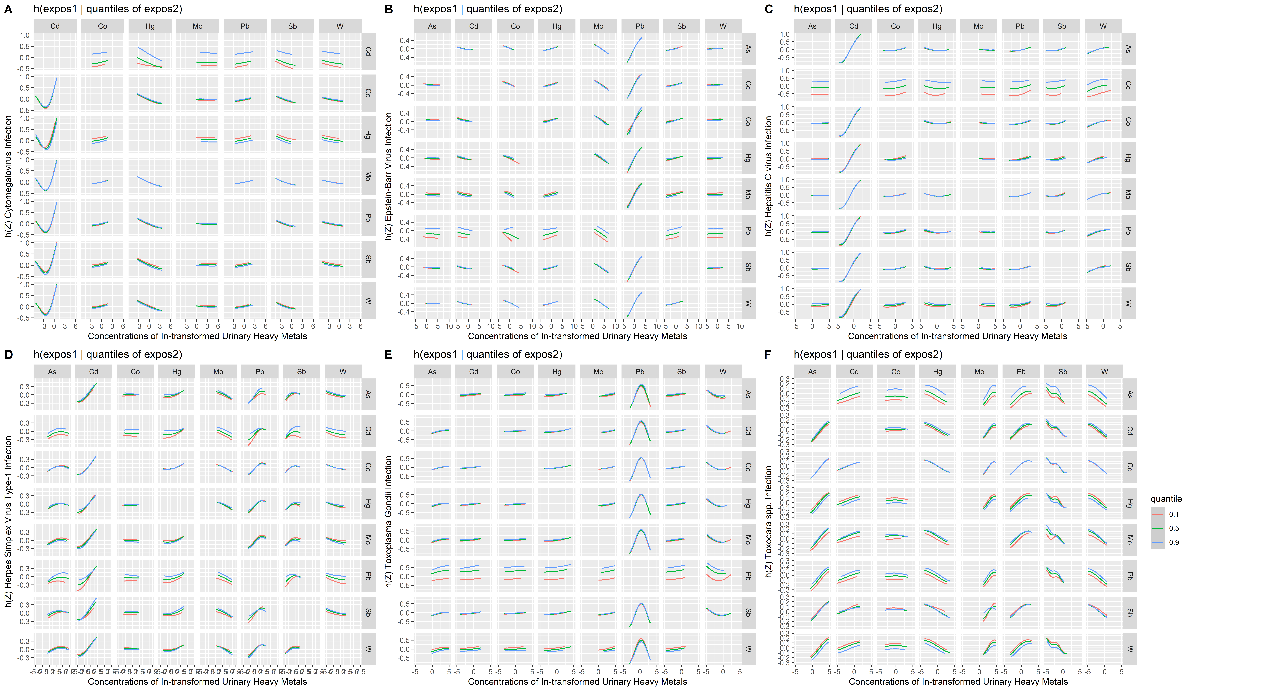


**Figure S3.** Bivariate exposure-response functions for every two metals on Cytomegalovirus infection (A), Epstein-Barr Virus infection (B), Hepatitis C Virus infection (C), Herpes Simplex Virus Type-1 infection (D), *Toxoplasma gondii* infection (E) and *Toxocara spp.* infection (F). h(Z) can be interpreted as the relationship between heavy metals and persistent infections.


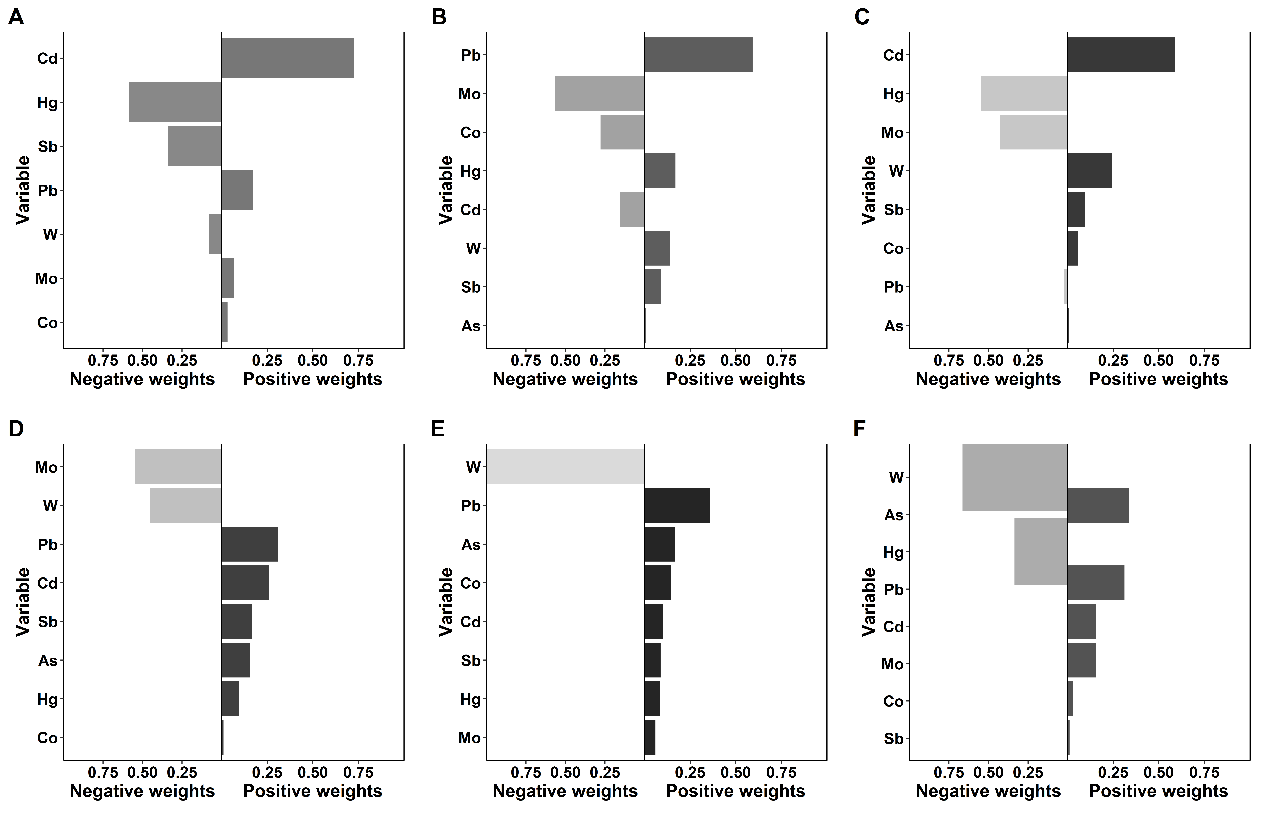


**Figure S4.** Qgcomp index weights of each heavy metals for the risks of persistent infections. The model was adjusted for gender, age, race, education level, poverty, BMI, serum cotinine, smoking status and alcohol consumption. Cytomegalovirus infection (A), Epstein-Barr Virus infection (B), Hepatitis C Virus infection (C), Herpes Simplex Virus Type-1 infection (D), *Toxoplasma gondii* infection (E) and *Toxocara spp.* infection (F).
